# Three different methods for ZnO-RGO nanocomposite synthesis and its adsorption capacity for methylene blue dye removal in a comparative study

**DOI:** 10.1186/s13065-025-01381-w

**Published:** 2025-01-18

**Authors:** Safaa A. Hussein, Gharib M. Taha, F. A. Adam, Marwa A. Moghazy

**Affiliations:** 1 Environmental Applications of Nanomaterial’s Lab., Department of Chemistry, Faculty of Science, Aswan University, Aswan, 81528 Egypt; 2 Department of Chemistry, Faculty of Science, Aswan University, Aswan, 81528 Egypt

**Keywords:** ZnO-RGO composite, Leidenfrost green method, Precipitation, Physical mixing method, Adsorption, Methylene blue

## Abstract

Water is one of the vital needs of life. However, due to rapid industrialization, urbanization and lack of awareness, the world population now facing the threat of water shortage. To ensure that future living conditions are preserved, it is crucial to reduce water pollution and protect the ecosystem. Zinc oxide- reduced graphene oxide (ZnO-RGO) nanocomposite is used in this study as an adsorbent for the adsorption of methylene blue (MB) dye from an aqueous solution. An easy strategy was used for the synthesis of reduced graphene oxide nanoparticles (RGO), Zinc oxide nanoparticles (ZnO) and ZnO-RGO nanocomposite. The synthesis of reduced graphene oxide (RGO) was accomplished through the exothermic reaction of a modified Hummer's method. In a novel approach, zinc oxide nanoparticles (ZnO NPs) were synthesized using the green Leidenfrost technique. This study presents a comparative investigation of ZnO-RGO nanocomposite synthesis employing both green and chemical methods. Three distinct approaches were utilized to prepare the ZnO-RGO nanocomposite: (1) the innovative Leidenfrost green method for composite A1, (2) a chemical precipitation method for composite A2, and (3) a physical mixing sonication method for composite A3. This research marks the first application of the Leidenfrost technique in the synthesis of ZnO-RGO nanocomposites, contributing to the growing body of knowledge in this field. X-ray diffraction (XRD), Burnauer-Emmett-Teller (BET), Fourier transform infrared (FTIR), Zeta potential, transmittance electron microscope (TEM) and scanning electron microscope (SEM) analyses are conducted for synthesized sample characterization. Comparing the XRD patterns of the three synthesis methods, it is notable that the intensity peaks of composite A3 were the highest when ZnO was synthesized using a green method, indicating a higher degree of crystallinity. FTIR analysis approves that combining ZnO with RGO affects the functional groups of the three nanocomposite surfaces. The SEM analysis shows ZnO NPs and RGO sheets are incorporated together. In the case of A1 composite sharp angles make a flower shape was observed due to the unique synthesizing method. The surface area for A2 composite is the highest (7.29 m^2^/g) compared with A1 (2.91 m^2^/g) and A3(1.90 m^2^/g). A comparison study is made among the three nanocomposites for MB dye removal. The effect of adsorbent dose, pH, contact time and initial dye concentration on dye adsorption has been studied. The results show that A1 and A2 nanocomposites removed 85.5 and 87.5% of MB at the optimum adsorbent dose of 0.15 g/100 ml at pH8 and A3 removed 95% of MB at the optimum dose of 0.1 g/100 ml at pH 2. All three composites exhibited adherence to the Langmuir isotherm model, with correlation coefficients (R^2^) of 0.9858, 0.9904, and 0.9959 for A1, A2, and A3, respectively. Kinetic study results demonstrated that the pseudo-second-order model best described the adsorption process for all three composites, yielding R^2^ values of 0.9998, 0.9988, and 1.0000 for A1, A2, and A3, respectively. The A3 nanocomposite shows the highest adsorption capacity (104.5 mg/g) compared to the other composites (87.7 and 97.5 mg/g for A1 and A2, respectively). Desorption experiments revealed that the dye removal percentages varied with the ratio of the ethanol–water mixture used. Absolute ethanol achieved a 90% removal compared with 1:1 and 1:2 aqueous ethanol solutions (87.5% and 80%, respectively).

## Introduction

Industrial pollutants often make water sources unsuitable for irrigation and life demands. The universe is full of cancer-causing substances because of increasing water contamination at a steady rate. Fast action is required to develop a potential and effective water management technology. Nowadays, everyone is concerned about water management due to the rising freshwater demand. Water sources are contaminated because of the discharge of various items such as colored pigments, pesticides, herbicides, diseases and heavy metals [1]. It is known that dye pollution affects human health, including the appearance of skin diseases and digestive problems that may increase the risk of cancer [2, 3]. One of the largest challenges is dye removal from the manufacturing and textile industry effluent [4]. These colored impurities are classified as anionic, cationic, or nonionic [1]. Methylene blue (MB) is one of these cationic-colored and most popular dyes. MB is frequently employed as a coloring and disinfectant in dyestuffs, rubbers, medicines, insecticides and varnishes [5]. In addition, it is used in coloring paper, wool, silk and hair coloring [6, 7]. MB dye is a highly toxic dye that is considered a source of several health problems such as respiratory disease, severe headaches, nausea, vomiting, stomach pain, chest pain, mental confusion, as well as a burning feeling if it is breathed or ingested [8, 9].

To effectively remove pollutants from wastewater, various techniques have been established. OF these, coagulation [10], sedimentation [11], filtration [12], chemical purification [13], reverse osmosis [14], photocatalytic degradation [15, 16], ion exchange [17], biological treatments [18] and other techniques were noted for water remediation [1]. The selection of water treatment techniques depends on flexibility and the lowest economic cost, so adsorption is considered an extremely widely used technique because it is a simple and inexpensive method [19]. Adsorption is a physicochemical surface interaction between the adsorbate and the adsorbent [20]. The adsorption method is affected by several factors, including temperature, the forces that interact between the adsorbent and adsorbate, the pH, contact time and concentration [21].

There are various adsorbents studied for MB dye removals such as crushed brick and cedar sawdust [22], garlic peel [23], bentonite [24], bamboo activated carbon [25], unburned carbon [26] and carbon nanotube [27]. Numerous studies have examined the effectiveness of graphene, graphene oxide (GO), reduced graphene oxide (RGO) and their composites due to their adsorption, oxidation and catalytic properties [28]. These compounds have significant pore volume, high conductivity and rich surface chemistry which make them favorable for the adsorption and catalysis of organic pollutants [29]. They have many applications for the adsorption of pollutants from an aqueous solution like the removal of heavy metal ions [30], oil spill [31], phenols [32], chlorophenols [33], pesticides [34], endocrine-disrupting compounds [35], photocatalysts [36], emerging pollutants [37] and microplastics [38].

To combine the properties of graphene and graphene oxide, RGO is prepared by thermal reduction of GO, where GO can regain graphene-like properties by reduction treatments that transform GO into RGO [39]. RGO is considered an intermediate of graphene and GO [40]. The region of the sufficient conjugated structure of graphene is considered an important point for RGO to adsorb organic dyes, particularly the aromatic structure. RGO possesses oxygen functional groups and surface defects (vacancies places), so RGO is better than GO and graphene in adsorption properties [41]. RGO gives favorable adsorption of MB due to the electrostatic interactions, π– π binding, and hydrophobic association [41–43].

Hummer’s method is considered one of the most popular ways for GO production. This technique has become extensively utilized for GO synthesis. It is a very quick and safe method where it was replaced sodium nitrate (NaNO_3_) and potassium permanganate (KMnO_4_) instead of nitric acid (HNO_3_) and potassium perchlorate (KClO_4_). It doesn’t produce harmful gases such as ClO_2_ (chlorine dioxide) or acidic fog. Now Hummer’s method is modified where it no longer uses NaNO_3_, so modified Hummer’s technique becomes more environmentally friendly [44].

ZnO is a favorable material due to its low cost, wide availability, eco-friendly nature, electrochemical activity and relatively broadband gap (3.37 eV) [45]. ZnO NPs have many applications as catalysis [46], photochemical capability [47], medical effects [48], fungicidal [49], antibacterial [50] UV filtration [51] and wastewater treatment [52]. Various techniques were used to synthesize ZnO NPs, in this study Leidenfrost method was used. Leidenfrost is considered a green nanotechnology that focuses on developing simple, novel and environmentally friendly ways to make nanoparticles [53]. The Leidenfrost effect occurs when a liquid comes into contact with a heated plate that has a temperature substantially higher than the liquid's boiling point creating an insulating vapor layer that prevents the liquid from rapidly boiling [53, 54]. The metal oxide forms when a drop of water is scattered on a hot surface at a surface temperature above the water boiling point, where the water molecules droplet ionized into H^+^ and OH^¯^. There is a negative charge inside the droplet because of the large number of hydroxyl ions, while there is a positive charge outside the droplet owing to the hydronium ion formation. Metal ion reacts with hydroxide ion forming a metal hydroxide which ends up into metal oxide [55].

"ZnO-RGO nanocomposite possesses remarkable properties; it combines the properties of both RGO and ZnO nanoparticles. It enhanced photocatalytic performance [56], energy storage capacity [57], sensing capability [58] and optoelectronic properties [59]. These attributes make them highly effective as adsorbents for the thorough removal of toxic metals and dyes from aqueous solutions. The increased surface area and diverse functional groups in graphene-based adsorbents contribute to their excellent adsorption potential for toxic metals and dyes. ZnO-RGO composite was synthesized using a new green method (Leidenfrost) that is different from traditional methods and is considered environmentally friendly which achieves the principle of sustainable development.

In this study, ZnO-RGO nanocomposites synthesized via a green method and chemical methods are compared for the first time. We examine the effect of the synthesis method on the resulting nanopowder properties and evaluate the efficacy of each composite in removing methylene blue (MB) dye from aqueous solutions, offering a comprehensive comparative analysis."

## Experimental

### Materials

In this study, all chemicals (graphite powder, Zinc nitrate-hexahydrate, potassium permanganate, Sulfuric acid (99%), phosphoric acid (85%), hydrochloric acid (99%) hydrogen peroxide (30%), methylene blue dye, ammonium hydroxide and potassium hydroxide) were of analytical grades used without additional purification.

### Synthesis of reduced graphene oxide

Using the modified Hummers method, pure graphite powder was used to prepare reduced graphene oxide (RGO). During this process, 27 ml of sulfuric acid and 3 ml of phosphoric acid (9:1 volume ratio) were mixed and stirred for a few minutes. While stirring, 0.225 g of graphite powder was added to the above solution. 1.32 g Potassium permanganate (KMnO_4_) was slowly added to the mixture. The obtained mixed solution was stirred for 6 h until the solution became dark green. To remove the excess KMnO_4_, 0.675 ml of hydrogen peroxide (H_2_O_2_) was added gradually and stirred for 10 min. This reaction is considered an exothermic reaction. The above mixture was centrifuged with 10 ml of concentrated hydrochloric acid and 30 ml of distilled water for 7 min. The residual was washed several times using distilled water. The result RGO was dried in an oven at 90 °C for 24 h. Functional groups in the GO are eliminated by a quick temperature change and GO is exfoliated to form RGO. Thermal shock is caused by an abrupt temperature change, which removes function groups from the GO lattice as water vapor, CO, and CO_2_. The primary cause of exfoliation is the pressure created by the evolution of gases between two stacked GO layers [60].

### Synthesis of ZnO

ZnO is prepared using the Leidenfrost method according to our previous work [61]. To a hot beaker (300 °C), a 50 ml of 10 mM solution of Zinc nitrate hexahydrate was added from a burette drop by drop to simulate the Leidenfrost effect by forming a vapor layer. Rapid vaporization processes take place in a very short time to create ZnO nanoparticles by heat convection, providing the required activation energy for the nucleation of nanoparticles**.**

### Synthesis of ZnO-RGO nanocomposite

#### First method (A1 nanocomposite)

For the first time, the ZnO-RGO nanocomposite is prepared using the Leidenfrost method. 50 ml of (0.01 M) Zn(NO_3_)_2_.6H_2_O was mixed with 50 ml (0.1 g) RGO. This mixture was sonicated for 10 min, then the mixture was added drop by drop (Leidenfrost effect) to a hot beaker (300 °C) to obtain the ZnO-RGO nanocomposite.

#### Second method (A2 nanocomposite)

50 ml of 0.01 M Zn(NO_3_)_2_.6H_2_O was mixed for an hour in an ice water bath using a magnetic bar. RGO solution was prepared by adding 0.0904 g of RGO to 100 ml of distilled water. The RGO solution was sonicated for one hour. Zn (NO_3_)_2_ solution was added to the RGO solution and mixed for 10 min, then 5.6 g of potassium hydroxide was added to the above mixture. The resulting solution was left for the night. The solution has become alkaline, so to make the solution pH 7, the precipitate was washed with distilled water during the filtration process. The obtained powder was dried in a 200°C oven for an hour**.**

#### Third method (A3 nanocomposite)

0.1g of RGO (50 ml) was sonicated for an hour. 0.4M zinc oxide nanoparticles solution was added slowly with stirring to the RGO solution. After stirring this mixture for 10 min, the suspension was allowed to age for 12 h before filtration. The obtained powder was dried for 3 h at 80°C**.**

### Characterization techniques

The synthesized samples were analyzed using an X-ray Diffraction (XRD) system from Germany, employing Cu Kα radiation at wavelength 0.154 nm. Field Emission Scanning Electron Microscopy (FE-SEM QUANTAFEG 250, Netherlands) was utilized at a voltage of 20 kV. Transmittance Electron Microscope (TEM), JEOL (JEM-HR-2100 ELECTRON MICROSCOPE, USA) was used for further examination. Fourier Transform Infrared analysis (FTIR) was conducted using a JASCO 3600 (Tokyo, Japan). These spectral measurements covered a range from 400 to 4000 cm^−1^ with a resolution of 2 cm^−1^, aiming to identify and analyze the functional groups present in the synthesized composites. The particle size distribution and charge characteristics of the synthesized composites were assessed using Zeta sizer Ver. 7.03 (temperature 25 °C, measurement position 2 mm). Additionally, (Burner–Emmett- Teller (BET) surface area analysis (Quanta CHROME NOVA2000 Series, UK) and a UV-1800 TOMOS spectrophotometer from China were employed at room temperature.

### Batch experiments

MB powder was dissolved in distilled water to get a stock solution of 500 mg/L. The standard working solutions were prepared daily by dilution of the stock solution. Batch experiments were carried out in 250 mL conical flasks containing 100 mL of standard solution. After adsorption equilibrium, the resultant solution was filtered using Whatman filter paper No. (1001 125), then the obtained solution was measured by the UV–visible spectrophotometer at λ_(max)_ = 664 nm.

The percentage removal (% removal) was calculated using the following equation:

1 $$\%\, Removal=\frac{{C}_{o}-{C}_{e}}{{C}_{o}}\times 100$$

where: C_o_ is the initial concentration; C_e_ is the final concentration in the solution.

The effect of pH on the adsorption of MB was studied in a pH range 2–10 for a 100 mL solution of MB (10, 20, 30, 50 and 100 mg/L). The pH of the solution was adapted to the required pH value using a 1M solution of HCl and NH_4_OH. The effect of the composite dose on the adsorption of the dye was examined with different dosages (0.02–0.2g). The contact time effect was studied in the range of 15–180 min at the optimum condition of pH, dye concentration and composites dose.

## Results and discussion

### Characterization

#### X-ray diffraction analysis (XRD)

**Fig. 1:**
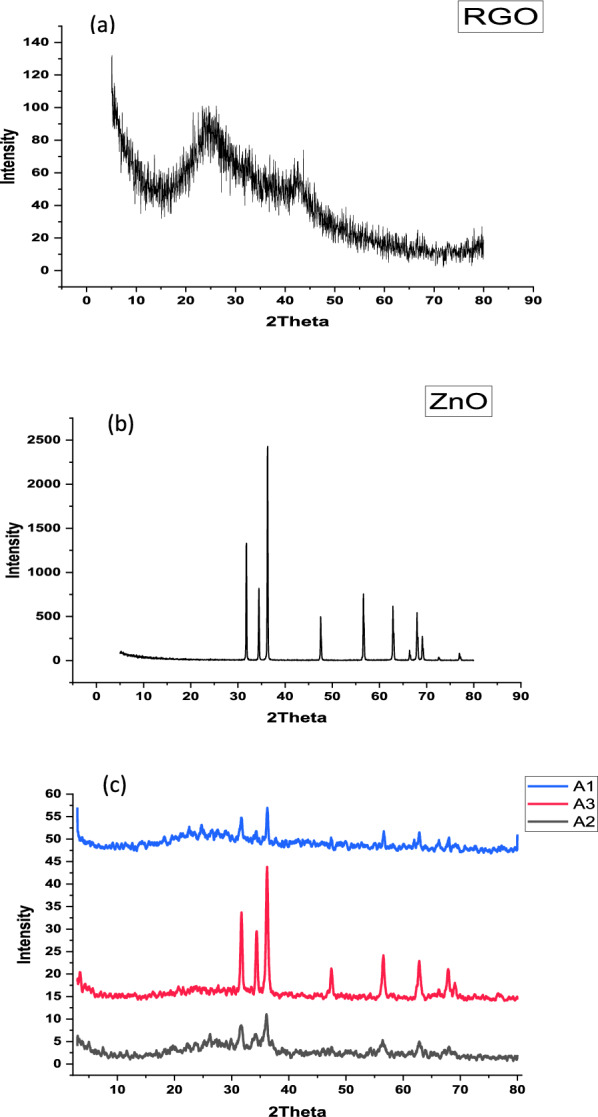
**a** XRD Pattern of Prepared RGO, **b** XRD Pattern of Prepared ZnO, **c** XRD Pattern of A1, A2 and A3

Figure 1a, b shows XRD analysis of RGO and ZnO nanoparticles. From the XRD pattern of the RGO, Fig. 1, two distinguished peaks can be observed the first which is the most intense peak at 2θ = 24.25º and the second, less intense at 2θ = 42.79º. The crystallite size of RGO was determined instrumentally to be 2.42 nm. For ZnO NPs, Fig. (1: b), multi-crystal with high crystallinity and pure phase of ZnO NPs was observed [61].

Figure 1c displays the XRD patterns of ZnO-RGO for the three prepared composites (A1, A2 and A3) which showed the combination peaks of ZnO and RGO. This Figure represents seven peaks were shifted to 2θ = 31.7°, 34.3°, 36.2°, 47.4°, 56.5°, 62.8° and 67.9° of ZnO for the three composites. As well, there is a low-intensity peak at 2θ = 28.8°, 28.7° and 24.2° for A1, A2 and A3, respectively which related to RGO [62]. The intensity of RGO peaks is low due to (1) its relatively low diffraction intensity compared to ZnO [63, 64] and (2) the dispersion of ZnO on the outer side of the RGO [65]. Notably, the intensity peaks of composite A3 were the highest where ZnO is prepared by a green method (Leidenfrost method), this indicates a higher degree of crystallinity. The crystallite size was determined to be 16.7, 33.8 and 65.2 nm for A1, A2 and A3, respectively.

#### Fourier transform infrared spectrometer (FTIR)

**Fig. 2:**
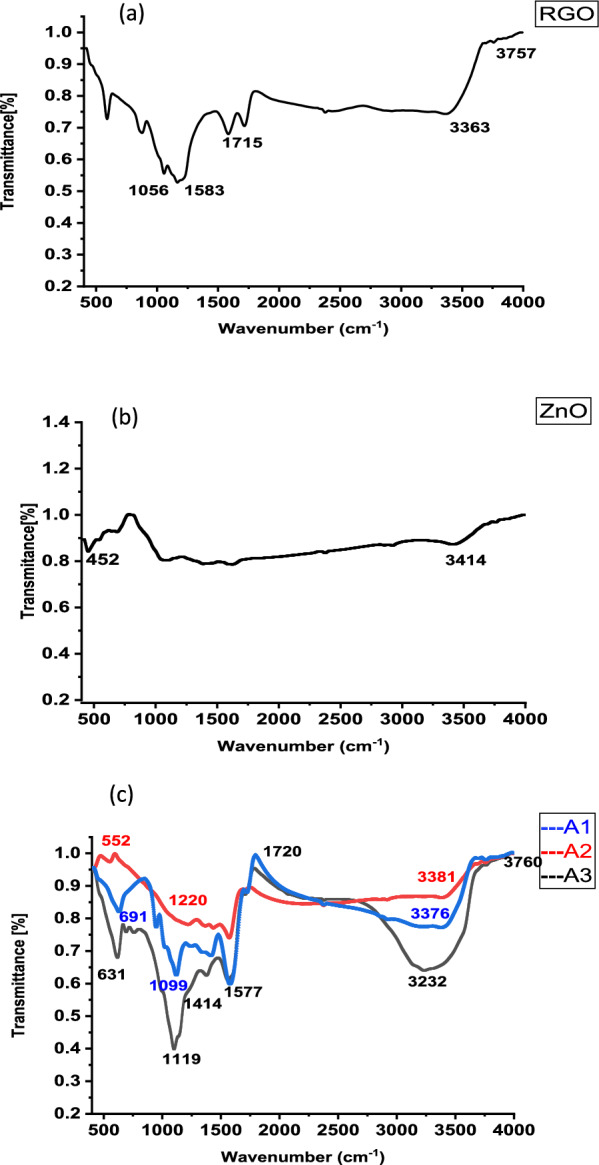
**a** FT-IR Spectrum of RGO, **b** FT-IR Spectrum of ZnO, **c** FT-IR Spectrum of A1, A2, A3

The FTIR analysis was studied for the RGO, ZnO and ZnO-RGO composites with a frequency range of 400 to 4000 cm^−1^. Figures 2a, b show an FT-IR of RGO and ZnO. For RGO, Fig. 2a, a typical broadband attributed to the − OH group was found at 3363 cm^−1^ which resulted from the adsorbed moisture [66]. The peak at 1715 cm^−1^ is assigned to the stretching C=O. Moreover, the peak at 1056 cm^−1^ refers to C–O stretching [67].

For ZnO, Fig. 2b, the peak at 452 cm^−1^ represented the stretching vibration of the Zn–O, in addition, a peak at 3414 cm^−1^ is related to the O–H stretching [61, 68–70].

Figure 2c shows the FTIR spectrum of the ZnO-RGO nanocomposites. A broad peak at 3232, 3381, 3376 and 1414 cm^−1^, which attributed to the O–H stretching vibration of the C–OH groups and water [71, 72]. The peaks at 1099.71, 1220.15 and 1119.67 cm^−1^ correspond to C–O bonds. The nanocomposite spectrum shows a strong transmittance band at 691, 552 and 631 cm^−1^ which is due to the vibrations of the ZnO bonds [70, 73] this confirms the ZnO-RGO Nanocomposite formation.

Comparing the peaks of RGO and the three nanocomposites, it was found that RGO has four main function groups that appeared at 3363, 1715, 1583 and 1056 cm^−1^ (OH, C=O of a carboxyl group, C=O of the carbonyl group and C-O, respectively). The same peaks with small shifts formed for A3 nanocomposite (3238, 1720, 1577 and 1119 cm^−1^) as well as the metal oxide peak at 631 cm^−1^. Alternatively, for A1 and A2 nanocomposites, only three peaks of RGO are noticed which are related to OH, C–O and C=O of the carbonyl group while the peak at 1715 cm^−1^ related to the carboxyl group is disappearing. The disappearance of the C=O of the carboxyl group in A1 and A2 nanocomposites means more reduction of RGO takes place [74, 75]. According to previous literature as the reduction process of RGO increases, the composite surface becomes more positive [74]. In contrast, the A3 nanocomposite surface accepted more negative charge compared with the RGO nanoparticle. This is because the A3 nanocomposite shows more broad and intensifying peaks for C=O of both carboxyl and carbonyl groups compared with RGO. In addition, four peaks of the OH group appeared at 3760, 3693, 3238 and 1414 cm^−1^ compared with RGO which gives two peaks only at 3757 and 3363 cm^−1^. The variation in the results between the three nanocomposites is related to the variation in their synthesis methods. For A1 and A2 composites, the ZnO NPs grew on the RGO, whereas for the A3 nanocomposite, the ZnO NPs mixed with RGO which accumulated on the surface of the RGO.

### Zeta potential analysis

**Fig. 3:**
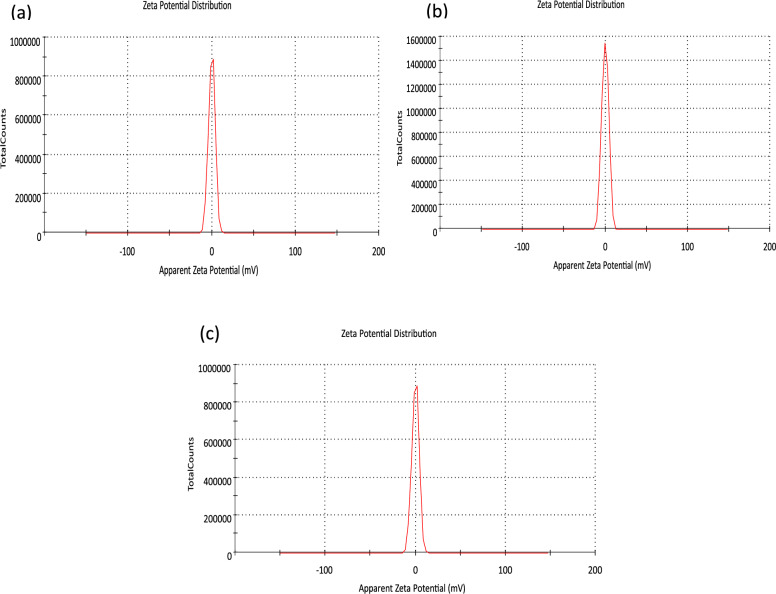
Zeta potential analysis (**a**) A1, (**b**) A2, (**c**) A3

The stability of the synthesized ZnO-RGO nanocomposites was assessed using zeta potential measurements, where a higher positive or negative value indicates greater nanoparticle stability [76]. The negatively charged surface of ZnO-RGO reflects the presence of oxygenated surface groups on RGO. The zeta potential values for A1, A2, and A3 were − 0.013, − 0.03, and − 0.012 mV, respectively, as shown in Fig. 3a–c, likely due to a reduction in oxygen functional groups [77]. Figure 3 further illustrates the consistent zeta potential distributions of A1, A2, and A3."

#### Scanning electron microscope (SEM)

**Fig. 4:**
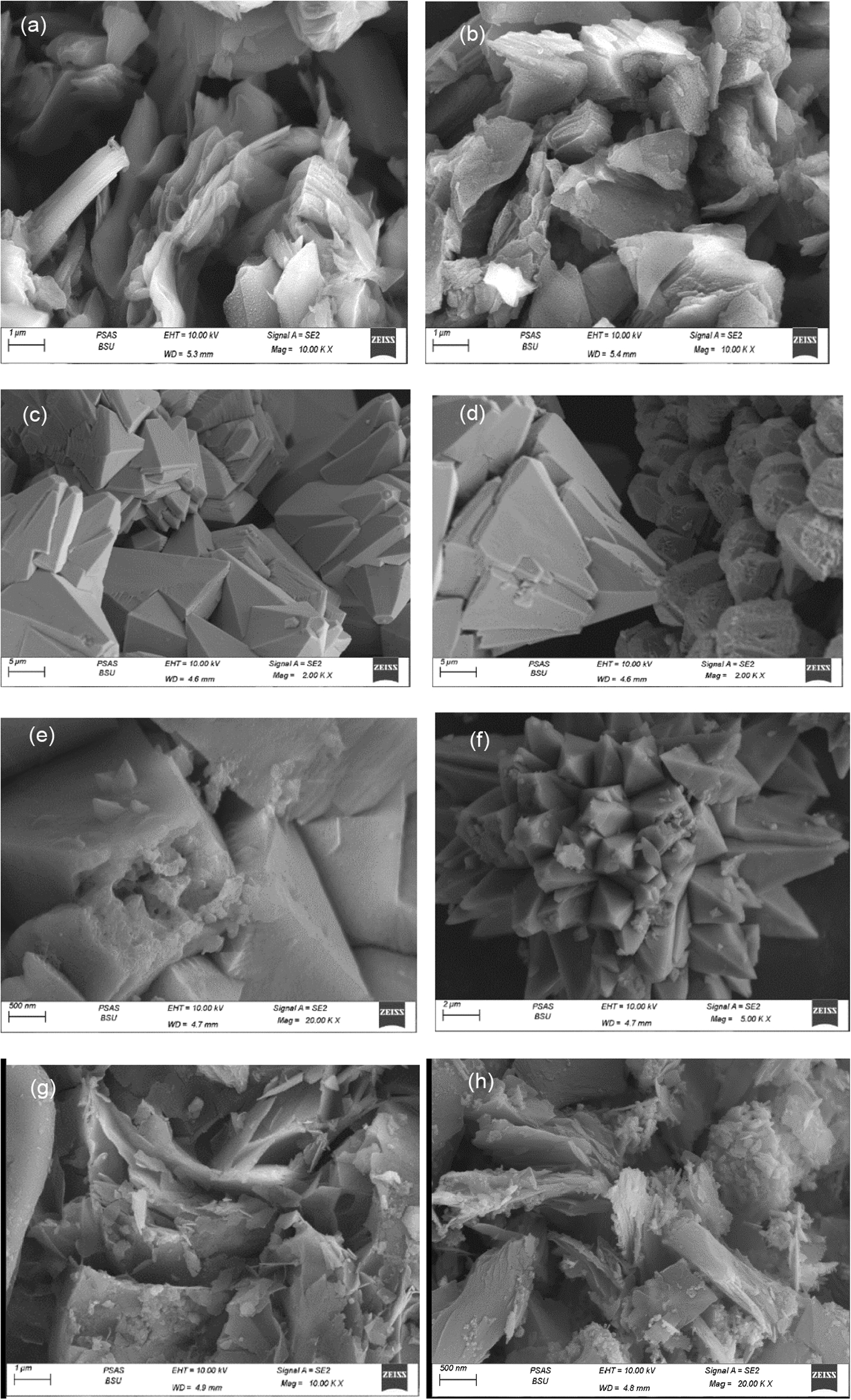

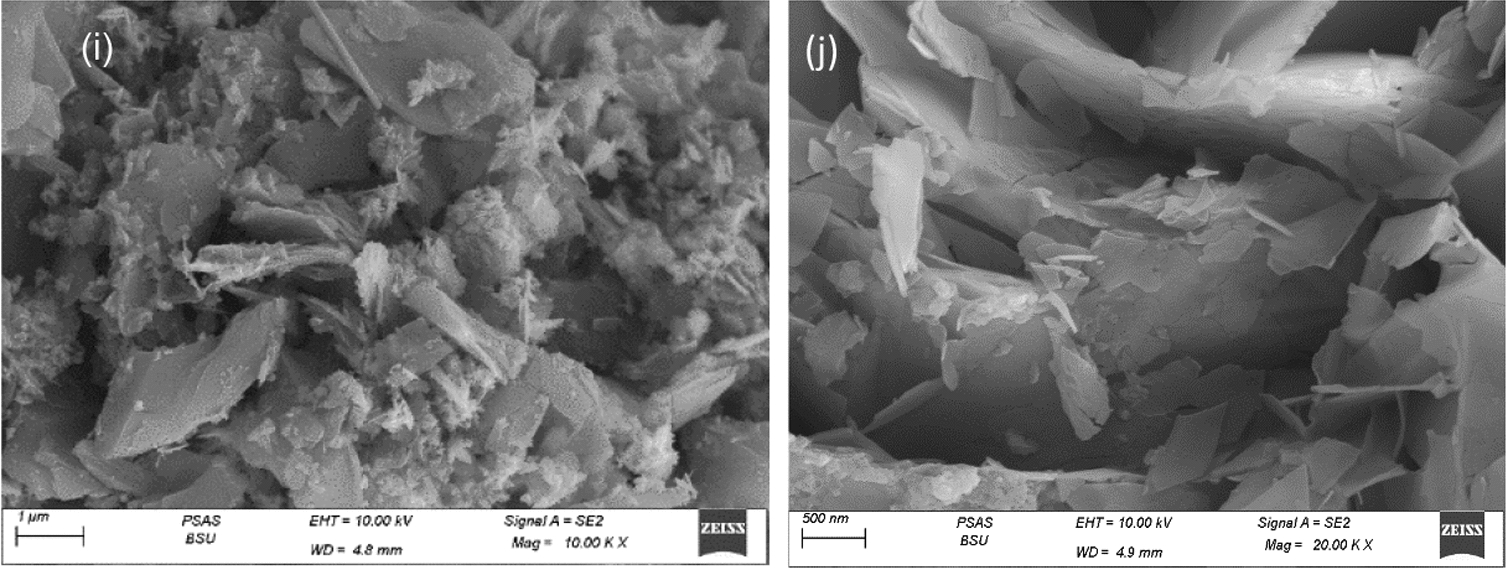
Scanning Electron Microscope (SEM) (**a** & **b**) for RGO, (**c**& **d**) for ZnO, (**e**& **f**) for A1composite, (**g**& **h**) for A2 composite, (**i**& **j**) forA3 composite

Figure 4a, b presents SEM images of RGO which appeared as a random aggregation of parallel thin sheets with different edges, wrinkled surfaces and folding [78]. Figure 4c, d represents the morphology of ZnO. They have six folded facets and are similar to the closely packed hexagonal bipyramid morphology [79]. Figure 4e, f shows the SEM images of the A1 composite. It exhibits a cross-linked flaky assembly as well as excellent dispersibility without cluster [80]. The structures randomly overlap one another and form sharp angles making a flower shape this is due to the unique method in which the composite was prepared [80]. Figure 4g–j show the SEM images of A2 and A3 composites, respectively. It shows ZnO nanoparticles and the RGO sheets are incorporated together [81].

#### Transmittance electron microscope (TEM)

**Fig. 5:**
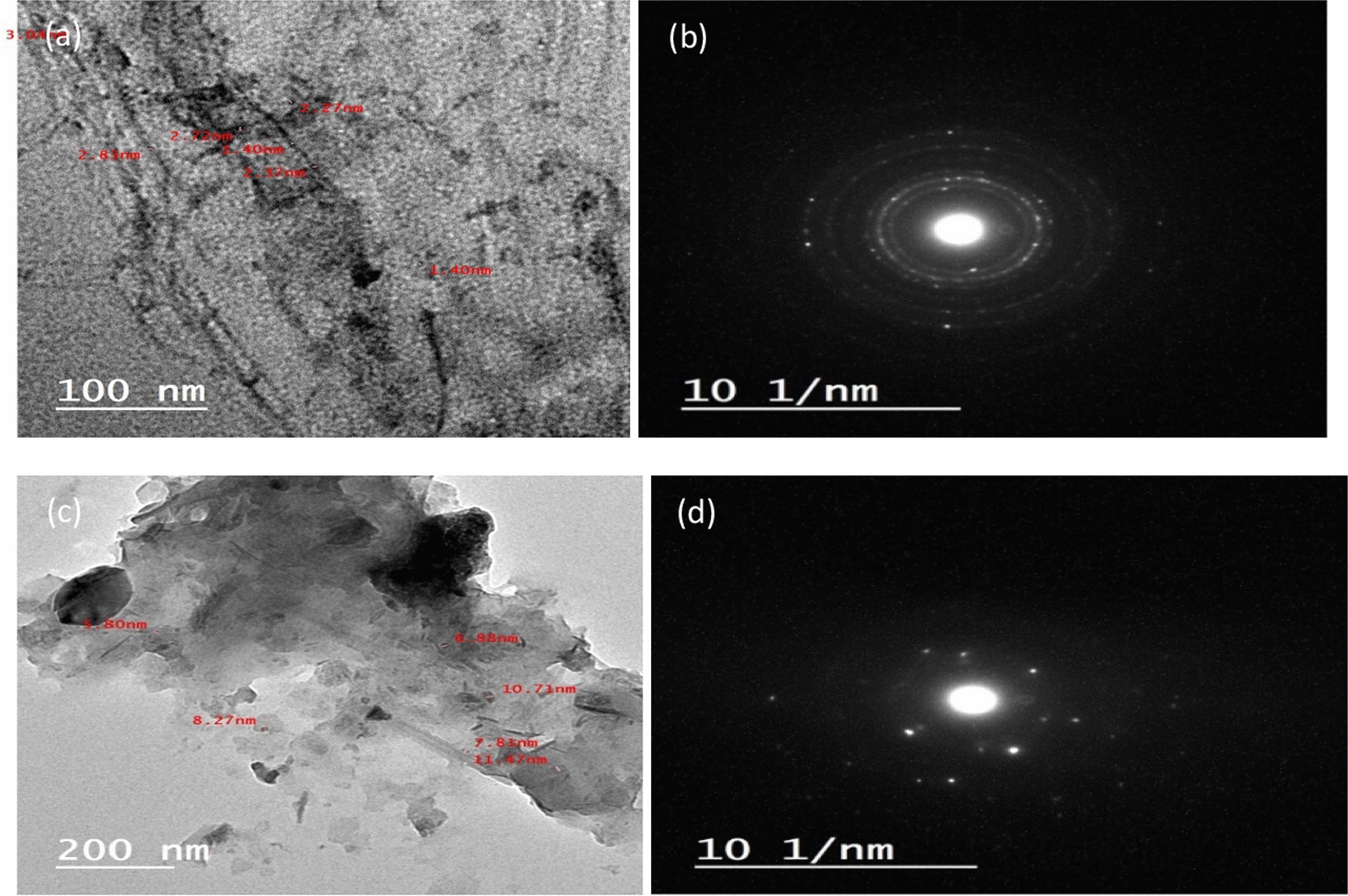
(**a**) TEM of RGO and (**b**) SAED pattern, (**c**) TEM for ZnO and (**d**) SAED pattern of ZnO

**Fig. 6:**
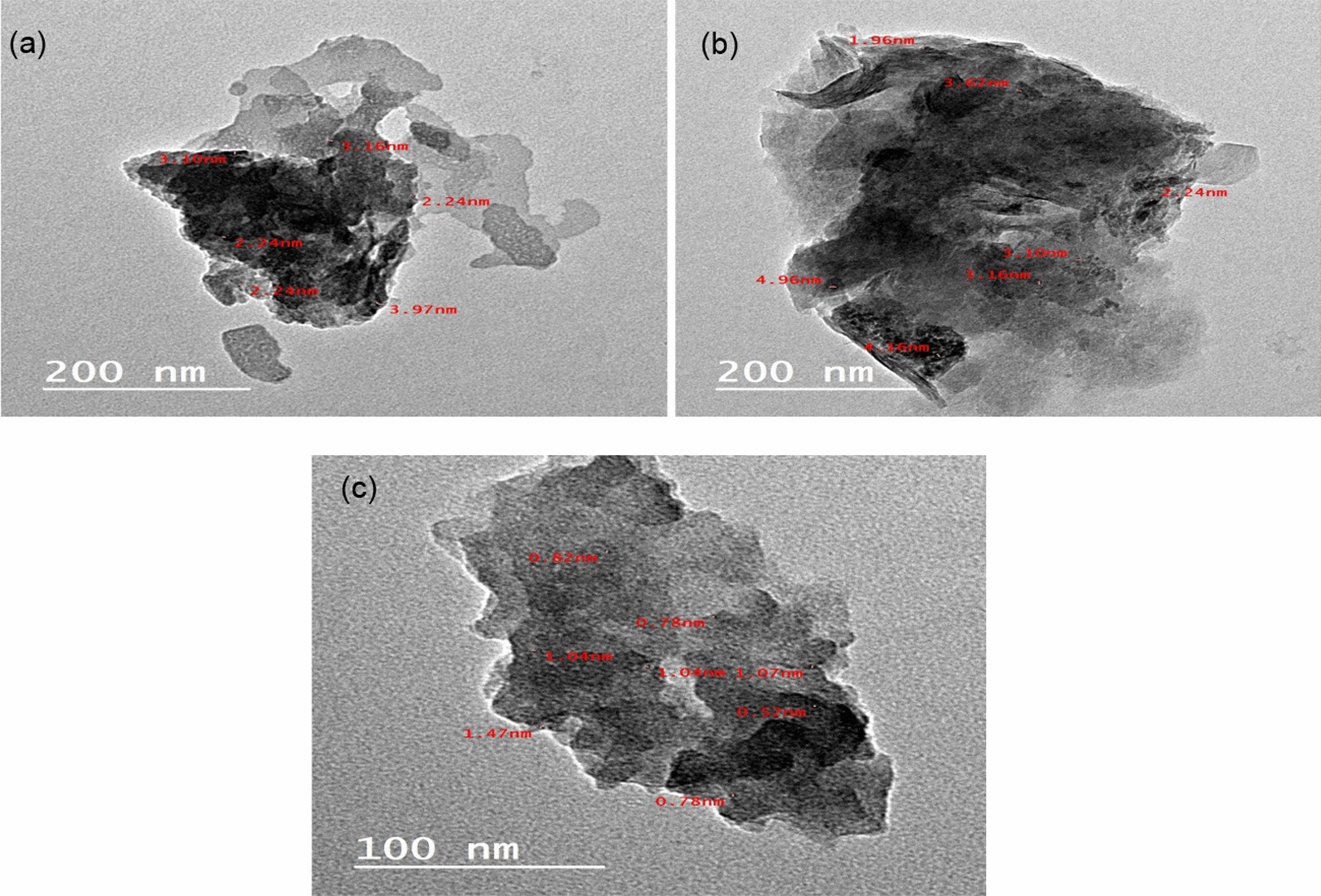
TEM images **a** A1 composite, **b** A2 composite, **c** A3 composite

The structure of synthesized samples was studied using the TEM technique. Figure 5a, b displays the TEM image of RGO which represents typical sheet-like silky waves and clumped structure, this Figure confirms the nano size of RGO in the range of (1.4–3.04 nm) [82]. The selected area electron diffraction (SAED) pattern of RGO displays well-defined diffraction rings and bright spots, indicating the crystalline nature of RGO [83–85]. For ZnO nanoparticles, Fig. 5c, d exhibits the formation of spherical-shaped nanoparticles which are agglomerated to form a cluster-like structure in the sample with particle size in the range of (5.8–11.47 nm) [86]. The SAED pattern of ZnO nanoparticles exhibits a characteristic ring diffraction pattern with some brighter, distinct spots, suggesting the presence of larger crystallites randomly oriented, though still in the nanometer range [87]. Figure 6a–c refers to the TEM images of A1, A2 and A3 composites, respectively. The ZnO nanoparticles are distributed randomly onto the surfaces of the RGO sheets. Additionally, the image shows that ZnO and RGO are in close contact. The particle size of A1 was in the order of (2.24–3.97 nm), for A2 (1.96–4.96 nm) and A3 (0.52–1.47 nm).

#### The surface area (BET) analysis

**Fig. 7:**
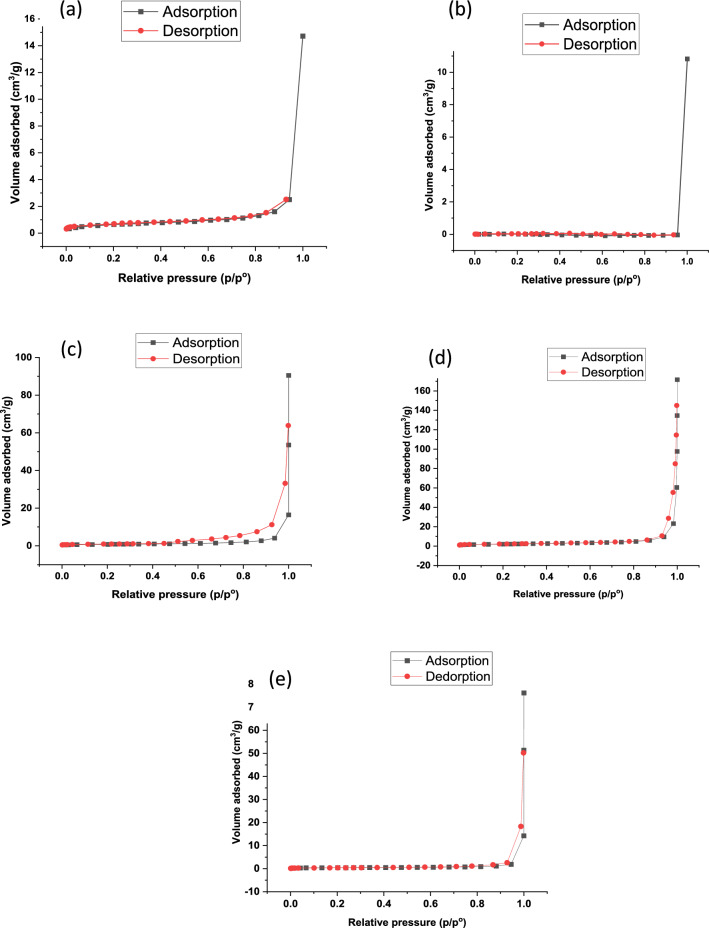
Nitrogen adsorption/desorption isotherm of **a** for RGO, **b** for ZnO, **c** for A1, **d** for A2, **e** for A3

The Burnauer-Emmett-Teller (BET) analysis is the most common one for measuring surface area by measuring the adsorption–desorption of nitrogen gas on the surface of the material [88]. The isotherm results of the RGO, ZnO and ZnO-RGO nanocomposites are presented in Fig. 7 and summarized in Table 1. It was observed that all nanocomposites (A1, A2 and A3) exhibited a type IV mesoporous isotherm with an H-1 hysteresis loop, according to IUPAC classification [89, 90]. It is noticeable that the BET-specific surface area of A2 is the highest among RGO, ZnO, A1 and A3 with a surface area of 7.29 m^2^/g. Conversely, the A3 composite gives the lowest surface area (1.90 m^2^/g) compared with the other composites. The A1 and A2 composites show the highest surface area which means the growth of ZnO NPs on RGO is effective and improves the properties of RGO and ZnO NPs individually. For the A3 composite, the surface area is lower than the RGO alone because the RGO pore is blocked with ZnO NPs because of the high concentration of ZnO that exists in this composite [91–93].

**Table 1:** Surface Areas, Pore Sizes and Pore Volumes for RGO, ZnO, A1, A2 and A3

Sample	Surface area (m^2^/g)	Pore size (nm)	Pore volume (cm^3^/g)
RGO	2.31	33.67	0.0194
ZnO	0.018	2935.4	0.0129
A1	2.91	30.75	0.0224
A2	7.29	35.17	0.0642
A3	1.90	39.09	0.0186

### Batch experiment

#### Effect of pH

Generally, pH value plays an important role in many adsorption processes. For example, the change in pH affects the way functional groups form on the adsorbent surface, which affects how adsorbent and adsorbate interact [5].

**Fig. 8:**
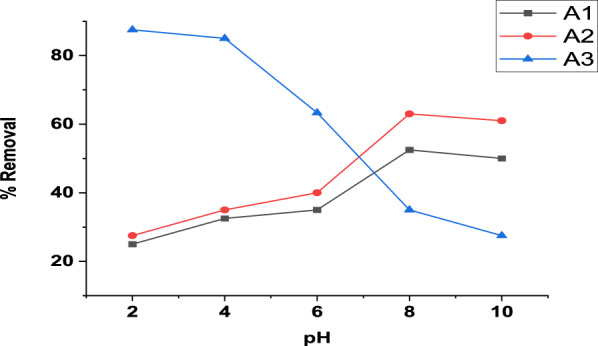
Effect of pH on MB adsorption (10 mg/L initial concentration, 0.02 g dose, 60 min and 380 rpm) using A1, A2, A3

The adsorption results for the removal of MB by the three composites adsorbents (A1, A2 and A3) at various pH values (2 to 10) under the experimental conditions (adsorbent dose 0.02 g /100mL, contact time of 60min and initial concentration 10 mg/L) were presented in Fig. 8. For A1 and A2 composites, the removal percentage increases from pH 2 (25% for and 27.5% for A2) to be maximum at pH 8 with 52.5 and 63% for A1 and A2, respectively. In contrast, the A3 composite shows high percentage removal at pH 2 with 87.5%, which decreases gradually to be minimized at pH 10 with 27.5%.

A1 and A2 composites achieve high removal at weak basic medium (pH 8) which is explained by the fact that the pH affects the adsorbent surface as follows: (a) below pH 4, the solution is intense with H^+^ ions which adsorbed together with the cationic dye molecules. (b) The second region (pH above 6) is practically neutral and only cationic dye molecules interact with the surface of the composites. (c) The third region (pH 10–11) has excess oxygen, OH^−^ ions, which interact with the cationic dye molecules, keeping them suspended in the solution [94]. Furthermore, the growth of ZnO on RGO leads to a decrease in ZnO PZC (pH_PZC_ = 9.07) to be neutral (pH_PZC_ = 7.2–7.7) for the ZnO-RGO composite [91, 95–99]. This means above the PZC the surface has a negative charge resulting in an electrostatic attraction force between the A1 and A2 composites and the positively charged MB cationic dye at pH 8.

In contrast, the optimum value for A3 at pH 2 (very acidic medium) where the percentage removal was 87.5%. This is attributed to two reasons: First, from BET analysis, the A3 composite surface pore is blocked by ZnO nanoparticles and in a very acidic medium (pH = 3) ZnO dissolved and transferred to the ionic state Zn^2+^ [91, 100]. This means at pH 2 an activation process takes place for the surface by dissociation of ZnO leading to more empty pores for MB dye adsorption. Increasing pH results in decreasing the adsorption percentage because of the presence of ZnO NPs on the RGO surface which hinders the adsorption of the dye. Secondly, from the FTIR analysis, Fig. 2c, the A3 composite contains more oxygenated function groups with higher intensity and broader than RGO making the composite surface has a negative charge and become more acidic. Increasing these groups (C–O, C=O and OH) leads to a negative value of zeta potential and reduces the PZC of the surface to be more acidic, where these groups are responsible for the acidity properties of the surface [74, 101]. This means the A3 composite PZC is below the PZC of RGO (RGO PZC = 3.5 [95]) so at pH lower than 3.5 the A3 surface has a negative charge facilitating the electrostatic attraction with cationic MB dye.

Comparing the three composites, A3 gives the highest removal percent of the MB dye, while A2 shows the lowest removal.

The results of A1 and A2 composites are in agreement with Mittal, et al., [102, 103], Mijinyawa, et al., [104], Ning, et al.,[105], Sarmah and Karak, [106], Sarkar, et al., [107], Yu, et al., [108] and Zhao, et al., [109] whose found that MB dye removed at pH 8. Otherwise, other studies found that the high adsorption capacity of MB dye takes place in an acidic medium [110–112].

#### Effect of adsorbent dose

**Fig. 9:**
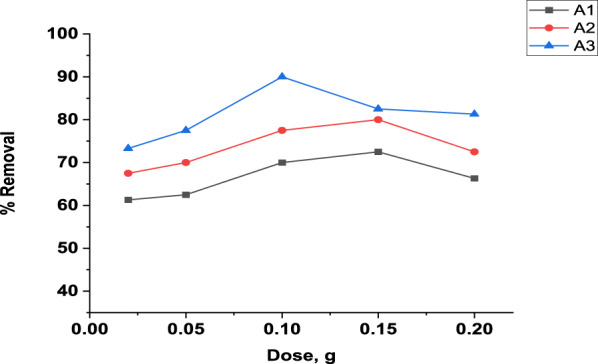
Effect of dose toward MB adsorption (10 mg/L initial concentration, 60 min and 380 rpm) using A1and A2 at pH = 8, A3 at pH = 2

The impact of MB dye removal using the three adsorbents at different dosages 0.02, 0.05, 0.1, 0.15 and 0.2 g/100 mL under the experimental conditions was studied. From Fig. 9, the general trend for the three composites is identical. Increasing the dose amount results in increasing the percent removal of MB dye to be maximum at 0.15 g for both A1 and A2 (72.5% and 80%, respectively) and 0.1 g for A3 (90%). Instead, by increasing the dose above 0.15g for A1 and A2 composites and 0.1g for A3 composites, the removal percentage decreases gradually. This is explained by the fact that increasing the adsorbent dose increased the number of active sites on the surface [113]. After equilibrium, the removal percentage decreased because of the saturation of the adsorbent surfaces, where all active sites are fully exposed and used. Also, at high doses, the adsorbent particles collide frequently, resulting in fewer active sites exposed and occupied by MB dye [114].

#### Effect of shaking time

**Fig. 10:**
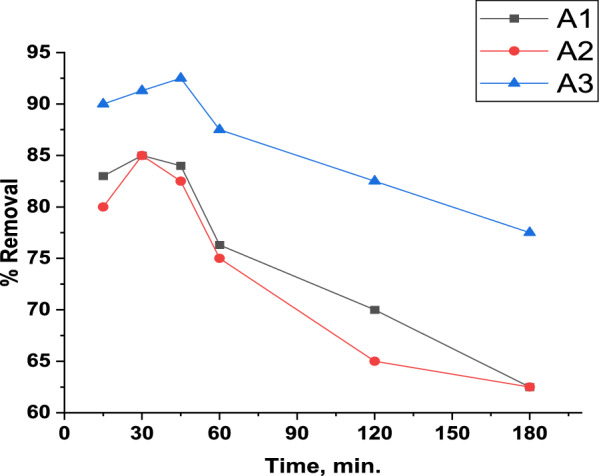
Effect of time toward MB adsorption (10 mg/L initial concentration, 0.15 g and pH = 8 for A1 and A2, 0.1 g and pH = 2 for A3 and 380 rpm)

Determining the contact time is essential for determining the time required for the adsorption process to reach equilibrium. The studied interval time was in the range of 15, 30, 45, 60, 120 and 180 min. The results are represented in Fig. 10. The graph reveals a rapid adsorption equilibrium time takes place for the three composites. Composites A1 and A2 show nearly similarity in results where, the removal increased from 15 to be maximum at 30 min with 85% and 85.9% for A1 and A2, respectively. For the A3 composite, the removal was raised from 15 to 45 min with 92.5% removal. For the three composites after equilibrium, the adsorption percentage goes down with increasing interval time to be the lowest at 180 min. The findings show that adsorption is a fast process and the nanocomposites have a high rate of adsorption due to their large exterior surface area and potential for rapid site occupancies, as well as having single-atom layers that are advantageous for quickly attracting dye molecules [98].

#### Effect of concentration

**Fig. 11:**
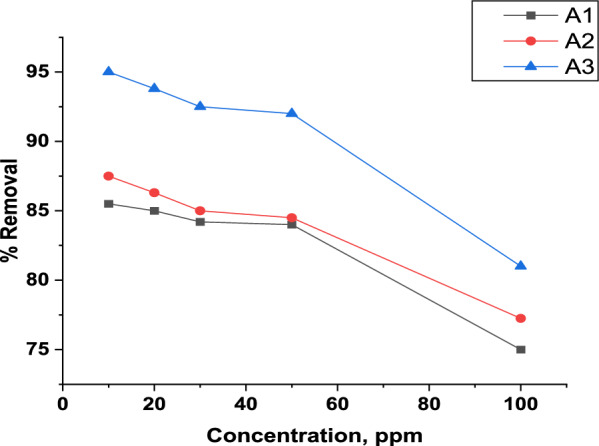
Effect of concentration toward MB adsorption (0.15 g, pH = 8, 30 min for A1 and A2, 0.1 g, pH = 2, 45 min for A3 at 380 rpm)

The impact of MB dye solution concentration on the adsorption capacity was evaluated in the range of 10- 100 mg/L. Figure 11 illustrates that there is a reverse relation between concentration and removal percentage; where the increase in concentration from 10 to 100 mg/L results in a decrease in the adsorption of the MB dye for the three composites**.** The highest removal for MB was obtained at 10 mg/L with removal percentages 85.5%, 87.5% and 95% for A1, A2 and A3, respectively. This may be explained by the fact that at lower dye concentrations the active sites are sufficient to adsorb the dye molecules but at a high dye concentration these active sites become saturated with dye molecules [115]. Finally, A3 gives the highest removal percentage for all studied concentration ranges compared with A1 and A2 composites.

**Fig. 12:**
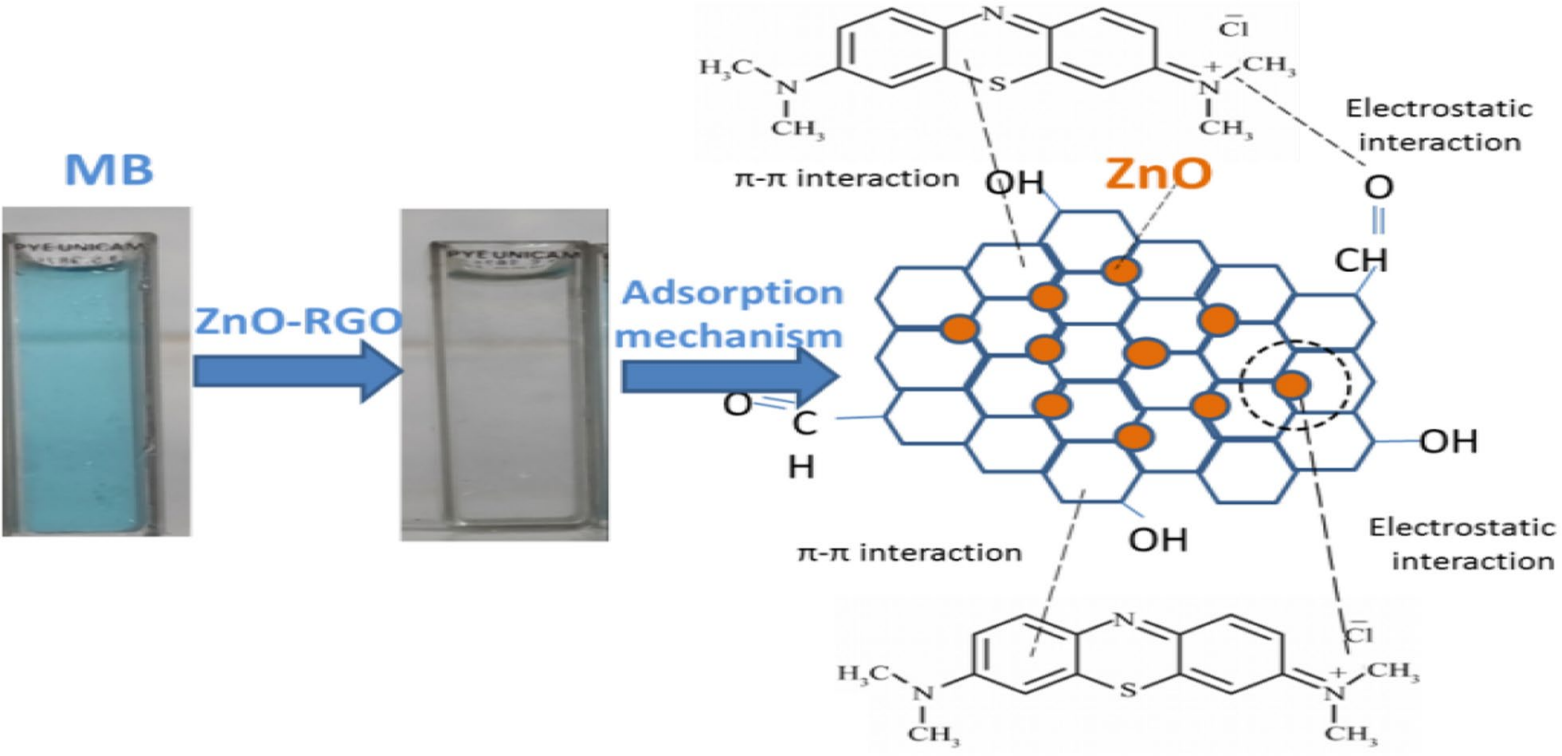
The mechanism of the adsorption process

Figure 12 illustrates the proposed adsorption mechanism of the ZnO-RGO composite for MB. The ZnO-RGO composite facilitates favorable adsorption of MB through hydrogen bonding, π-π interactions, and electrostatic attraction. In an aqueous solution, MB exists as positively charged ions, while ZnO-RGO tends to acquire a negative charge in water, enhancing the electrostatic attraction between the dye and the adsorbent surface.

#### Adsorption isotherm

Several mathematical models have been applied to describe equilibrium studies for the adsorption process. Langmuir and Freundlich models are the most applicable models for adsorption systems. Freundlich model can describe adsorption on heterogeneous rough surfaces and the interactions within the adsorbed molecules [41]**.** The Freundlich isotherm is introduced as an empirical Model (Eq. 2):

2 $$Log\, {q}_{m}=Log\, {K}_{f}+\frac{1}{n}Log\,{ C}_{e}$$

where *q*_*e*_ the equilibrium capacity of dye on adsorbent (mg/g), C_e_ is the equilibrium concentration of heavy metals or dye solution (mg/L), K_f_ is the Freundlich isotherm constant and (n) is the adsorption intensity. K_f_ and 1/n can be calculated from the intercept and slope of the linear plot of Log q_e_ against Log C_e_ [116].

According to the Langmuir adsorption isotherm, adsorption only occurs on homogenous surfaces [41]. The linearized form of the Langmuir equation is described by equation (3):

3 $$\frac{{C}_{e}}{{q}_{m}}=\frac{{C}_{e}}{{q}_{m}}+\frac{1}{{q}_{m}{K}_{L}}$$

where C_e_ is the equilibrium concentration of dye solution (mg/L), q_m_ is a monolayer adsorption capacity of the adsorbent (mg/g), K_L_ is the Langmuir constant related to the energy and affinity of binding sites of adsorption (L/mg). The q_m_ and K_L_ can be calculated from the intercept and slope of the linear plot of C_e_/q_e_ against C_e_.

The linearized form of the Temkin equation is expressed as:

4 $${q}_{e}=B\, lnK+B\, ln{C}_{e}$$

where B = RT/b, b is the Temkin constant related to the heat of sorption. Q_e_ (mg/g) and C_e_ (mg/L) are the amount of adsorbed dye per unit weight of adsorbent and un-adsorbed dye concentration at equilibrium, respectively. Therefore, a plot of q_e_ versus ln C_e_ is used to determine the constant B which is the constant related to the heat of sorption (J/mol) and K, is the Temkin isotherm equilibrium binding constant [117].

The linearized D-R equation can be written as:

5 $$Ln\, {q}_{e}=\text{ln}\,{q}_{m}-\beta {E}^{2}$$

where β, is a constant related to the adsorption energy (mol^2^/kJ^2^), q_m_ is a constant that indicates the sorption degree characterizing the sorbent (mg/g) and E is the Polanyi potential, which can be obtained by following equation [118]:

6 $$E=RT\,\text{ln}(1+\frac{1}{{C}_{e}})$$

where R, is the ideal gas constant (R = 8.314 J/mol) and T is absolute temperature (K). By plotting ln q_e_ vs E^2^ it is possible to determine the value of β from the slope and the value of q_m_ from the intercept.

The mean free energy E (mol/kJ) of sorption can be estimated by using B values as expressed in the following equation:

7 $$E = \frac{1}{{(2\beta )^{1/2} }}$$

The magnitude of E may characterize the type of adsorption as chemical ion exchange (E = 8–16 kJ/mol) or physical adsorption (E < 8 kJ/mol) [119].

**Fig. 13:**
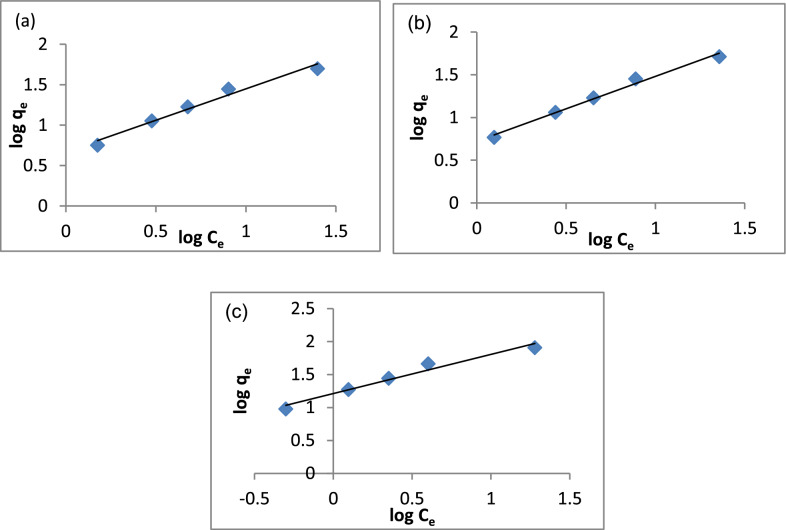
Freundlich isotherm model **a** A1, **b** A2, **c** A3

Figure 13a–c represents the Freundlich isotherm of A1, A2 and A3 composites. According to Eq. (2), the k_f_ is determined to be 4.70, 5.27 and 13.21 for A1, A2 and A3, correspondingly. The values of 1/n are summarized in Table 2. The value of 1/n between 0 and 1 indicates the adsorption intensity of a normal isotherm [120]. The R^2^ were 0.9858, 0.9896 and 0.9682 for A1, A2 and A3, individually.

**Table 2:** The Summary of Freundlich, Langmuir Isotherms, Temkin and Dubinin-Radushkevich Isotherm Models

Composite	Freundlich Isotherm			
	K_f_ (mg/g)	n	1/n	R^2^
A1	4.70	1.36	0.73	0.9858
A2	5.278	1.31	0.75	0.9896
A3	13.21	1.33	0.74	0.9682

Composite	Langmuir Isotherm			
	q_m_ (mg/g)	K_L_	R_L_	R^2^
A1	87.70	0.05	0.66	0.9760
A2	97.50	0.05	0.66	0.9904
A3	104.50	0.18	0.35	0.9959

Composite	Temkin Isotherm			
	B (J/mol)	b_t_ (J/mol)	K_t_ (L/g)	R^2^
A1	15.21	162.89	0.85	0.9463
A2	16.07	154.17	0.85	0.9472
A3	20.42	121.33	2.36	0.9687

Composite	Dubin-Raduskevich Isotherm			
	q_m_ (mg/g)	E (KJ/mol)	β (mol/kJ)	R^2^
A1	27.85	0.79	8*10^–7^	0.7345
A2	29.10	0.79	8*10^–7^	0.7606
A3	47.06	1.58	2*10^–7^	0.7734

**Fig. 14:**
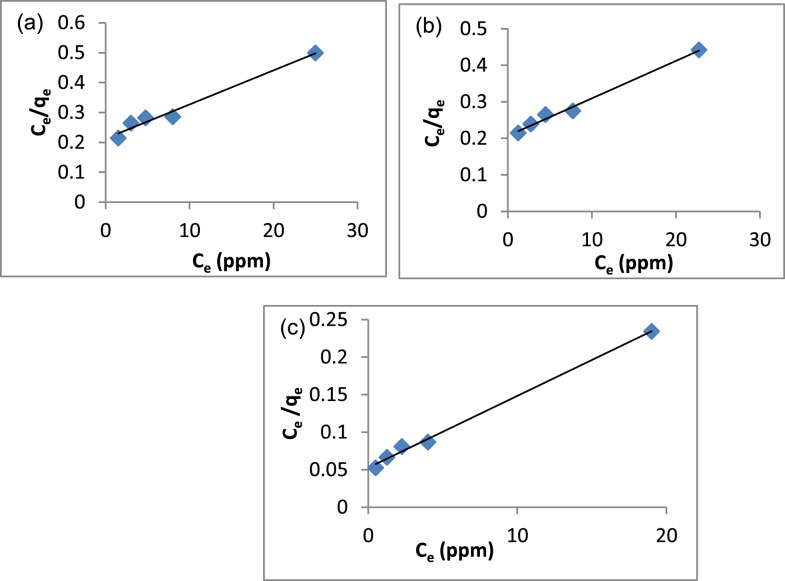
Langmuir Isotherm Model **a** A1, **b** A2, **c** A3

The Langmuir isotherms, (Eq. 3), were represented in Fig. 14a–c and Table 2. The adsorption capacities (q_m_) were determined to be 87.7, 97.5 and 104.5 for A1, A2 and A3, respectively. The correlation coefficients (R^2^) were 0.9760, 0.9904 and 0.9959 for A1, A2 and A3, correspondingly. These findings suggest that Freundlich isotherm applies to the three composites but compared with the R^2^ of Langmuir, the Langmuir model is more convenient for the three composites. Furthermore, from Table 2, it is noticed that the k_L_ < 1 which confirms the adsorption of MB on A1, A2 and A3 favors the Langmuir isotherm [120]. Comparing the q_m_ values of the three composites indicated that the A3 composite has high-capacity adsorption for MB dye compared with A1 and A2 composites.

**Fig. 15:**
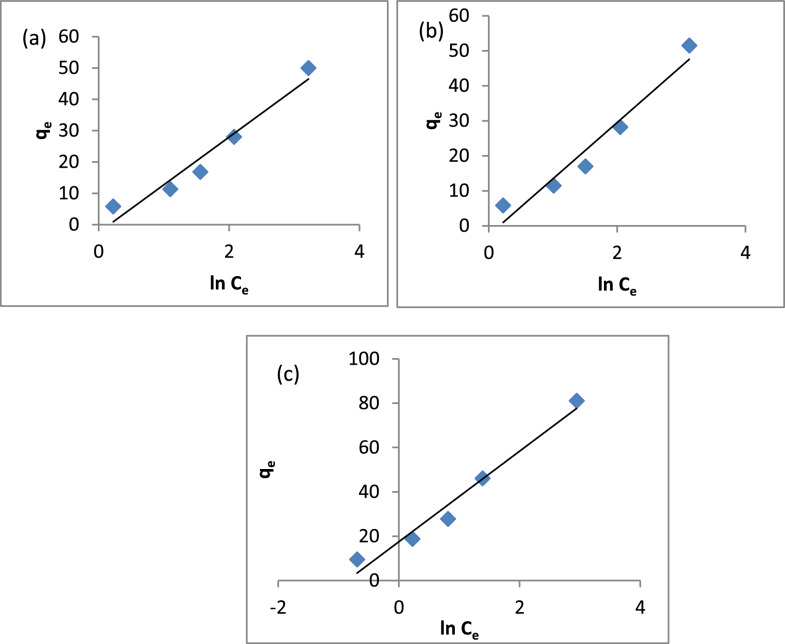
Temkin Isotherm Model **a** A1, **b** A2, **c** A3

**Fig. 16:**
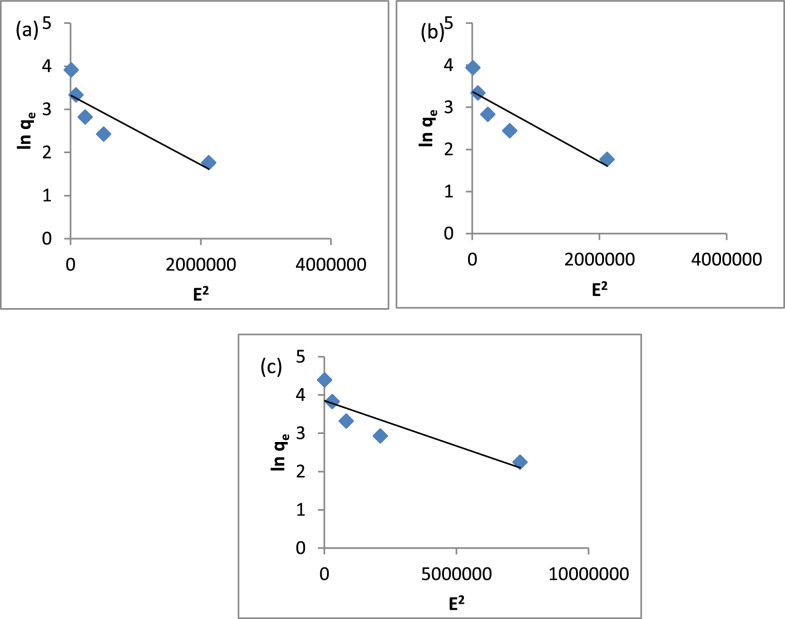
Dubinin-Radushkevich Isotherm Model **a** A1, **b** A2, (**c**)

Figure 15a–c shows the Temkin isotherm plots for the A1, A2, and A3 composites, with the values of B and K provided in Table 2. The B values were 15.21, 16.07, and 20.41, respectively, while the K values were 0.85, 0.85, and 2.35, correspondingly. Figure 16a–c presents the Dubinin-Radushkevich isotherm for A1, A2, and A3, with Table 2 indicating that the mean free energy (E) values are 0.79, 0.79, and 1.58 kJ/mol. These values suggest that MB adsorption on A1, A2, and A3 occurs via physical adsorption. Therefore, it can be concluded that physical adsorption plays a dominant role in the MB adsorption process on these three composites.

#### Adsorption kinetic studies

**Fig. 17:**
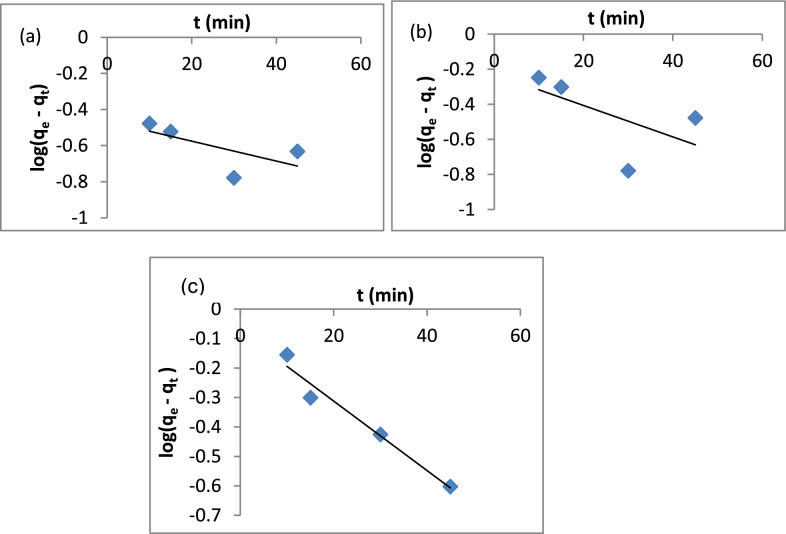
Pseudo-first order model **a** A1, **b** A2, **c** A3

The adsorption kinetic study provides facts about the adsorption rate, adsorbent performance and mass transfer mechanisms. There are three steps in the kinetics of adsorption mass transference. The first step is external diffusion, in which the adsorbate moves across the liquid film surrounding the adsorbent. The external diffusion is driven by the concentration dissimilarity between the bulk solution and the adsorbent surface. The second step is internal diffusion. Internal diffusion refers to the movement of adsorbate through the pores of an adsorbent. The third step is the adsorption of the adsorbate in the active sites of the adsorbent. The models of the pseudo-first-order and the pseudo-second-order are the most used [121]. The pseudo-first-order kinetic model is described by Eq. (8):

8 $$Log\left({q}_{e}-{q}_{t}\right)=Log{q}_{e}-\left(\frac{{K}_{1}}{2.303}\right)t$$

where, q_e_ and q_t_ are the adsorption capacities at equilibrium and time (mg/g), respectively. K_1_ is the rate constant of the pseudo-first-order [122]. A plot of log (q_e_-q_t_) vs t. Figure 17a–c gives a linear relationship with the slope k_1_ and intercept log q_e_ according to Eq. (8). The results in Table 3 show that k_1_ (min^−1^) for A1, A2 and A3 are 0.012, 0.02 and 0.027, respectively. The correlation coefficients (R^2^) are 0.4261, 0.3505 and 0.9641 for A1, A2 and A3, respectively.

**Table 3:** Summary of pseudo-first order, pseudo-second order, intra-particle diffusion and Elovich models

Composite	Pseudo-first order		
	K_1_ (min^−1^)	q_e_ (mg/g)	R^2^
A1	0.012	0.34	0.4261
A2	0.020	0.59	0.3505
A3	0.027	0.83	0.9641

Composite	Pseudo-second order		
	K_2_ (g mg^−1^ min^−1^)	q_e_ (mg/g)	R^2^
A1	0.82	5.65	0.9998
A2	0.33	5.62	0.9988
A3	0.16	9.38	1

Composite	Intra-particle diffusion		
	K_i_	C (mg/g)	R^2^
A1	0.04	5.41	0.5875
A2	0.09	5.03	0.5749
A3	0.12	8.49	0.9349

Composite	Elovich		
	α (mg/g min)	β (g/mg)	R^2^
A1	9.2*10^24^	11.29	0.6659
A2	1.4*10^5^	4.71	0.6406
A3	1.6*10^12^	3.59	0.9619

**Fig. 18:**
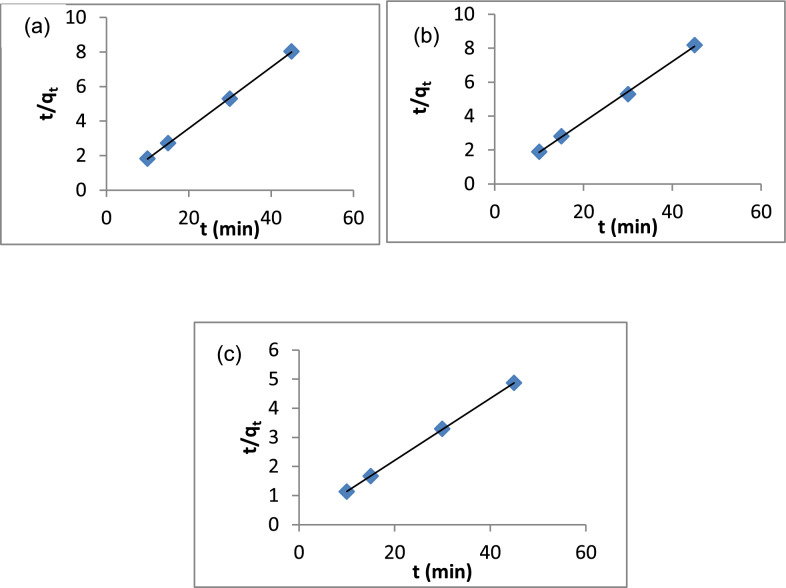
Pseudo-second order model: **a** A1, **b** A2, **c** A3

The linearized form of the pseudo-second-order kinetic model is represented as:

9 $$\frac{t}{{q}_{t}}=\frac{1}{{k}_{2}{q}_{e}^{2}}+\frac{1}{{q}_{e}}t$$

where, q_e_ and q_t_ are the adsorption capacities at equilibrium and time (mg/g), respectively. A plot of t/q_t_ vs. t. Figure 18a–c shows a linear relationship. The data in Table 3 shows the equilibrium adsorption capacity (q_e_) for A1, A2 and A3 are 5.65, 5.62 and 9.38, respectively. The correlation coefficients (R^2^) for A1, A2 and A3 are 0.9998, 0.9988 and 1, respectively. All the correlation coefficients (R^2^) of pseudo-second-order reaction kinetics are higher than the first-order reaction kinetics, indicating that the removal of MB using A1, A2 and A3 is well described by pseudo-second-order kinetics.

**Fig. 19:**
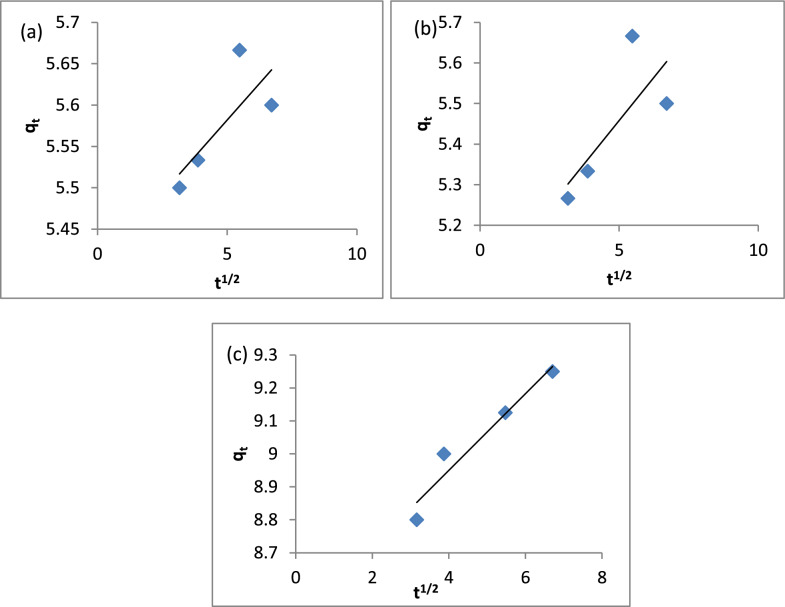
Intra-particle diffusion model: **a** A1, **b** A2, **c** A3

The intra-particle diffusion model is represented as [123]:

10 $$q_{t} = K_{i } t^{1/2} + C$$

where K_i_ (mg g^−1^ min^−1/2^) is the rate constant of the intra-particle diffusion model and C (mg/g) reflects the boundary effect. The intra-particle diffusion plots of q_t_ vs t^1/2^ are shown in Fig. 19a–c and the values are given in Table 3. The data in Table 3 shows that the C values for A1, A2 and A3 are 0.035, 0.085 and 0.12 mg/g, respectively.

**Fig. 20:**
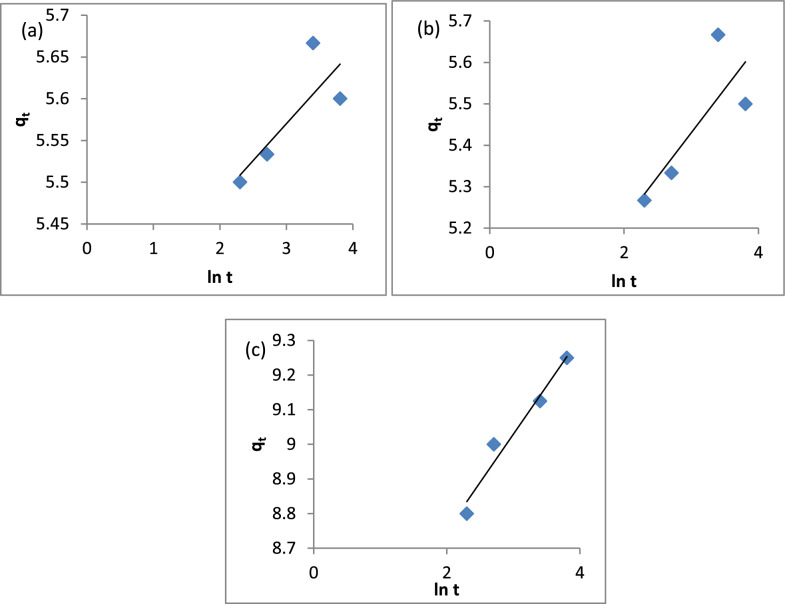
Elovich model: **a** A1, **b** A2, **c** A3

The Elovich equation was:

11 $${q}_{t }=\frac{1}{\beta }\text{ln}\left(\alpha \beta \right)+\frac{1}{\beta }\text{ln}t$$

where, α and β are the initial adsorption rate and the adsorption coefficient, respectively. The constants αand β can be calculated when q_t_ is plotted vs ln t [124]. The results are shown in Fig. 20a–c. The data in Table 3 shows the initial adsorption rate (α) for A1, A2 and A3 are 1.6*10^24^, 1.4*10^5^ and 1.6*10^12^, respectively. The adsorption constant (β) for A1, A2 and A3 are 11.29, 4.71 and 3.59, respectively.

Table 4 lists the batch experimental optimum condition and qm of this study in comparison to the results of other studies. It is noticeable that the pH values were between 5 and 10, which is nearly neutral, only A3 prefer the acidic medium. Comparing the three adsorbents, the ability of the adsorbent surfaces to remove MB is 87.7, 97.5, and 104 mg/g for A1, A2, and A3, respectively. Compared to A1 and A2, the q_m_ for A3 is very high.

**Table 4:** The maximum removal percent and capacity of Methylene Blue at different conditions using various adsorbents

Adsorbent	pH	Dose (g/L)	Time (min)	Conc. (ppm)	Removal %	q_m_ (mg/L)	Reference
Composite A1	8	1.5	30	10	85.5	87.7	This paper
Composite A2	8	1.5	30	10	87.5	97.5	This paper
Composite A3	2	1	45	10	95	104	This paper
ATRH/NaOH	Unbuffered	0.8	60	5	96	28.42	[125]
Hordeum Murinum	5.6	20	60	20	94	–	[126]
Fig leaf activated carbon (FLAC)	7	3.2	60	80	99.3	69.93	[127]
Fe_3_O_4_-PHEMAIA	11	1.5	15	20	–	16.129	[128]
CS-MgONP	7.28	0.47	-	19.37	94.5	163.87	[129]
Alg/Clin/Fe_3_O_4_	10	2	60	10	93.62	12.48	[130]
Iron impregnated nanocla	7	0.08	120	40	–	79.68	[131]
Musa paradisiaca stem	12	12	90	70	91.4	–	[132]
Cashew nut shell-based activated carbon	5	4.2	180	20	–	23.28	[133]
Kaolin	6	0.5	120	100	–	52.76	[134]
Enset (Ensete ventricosum midrib leaf, EVML)	5.07	2.5	60	10	–	35.50	[135]
Gnetum gnemon shell waste	10	–	25	100	–	35.58	[136]
Sugracan e bagasse (SCB)	–	2	–	25	–	25.25	[137]

#### Desorption of ZnO-RGO composite

The desorption process using volatile organic solvents was explored as an effective approach for dye removal [138]. The choice of solvent is crucial for efficient dye desorption; it should be affordable, environmentally friendly, and possess high dye solubility [139]. Ethanol was selected to regenerate the ZnO-RGO composite. For the desorption experiment, ZnO-RGO composite was immersed in 50 ml of absolute ethanol, 1:1 aqueous ethanol, and 1:2 aqueous ethanol for 10 min to facilitate MB dye desorption. A 10 ml sample of 10 mg/L MB dye was filtered, and the filtrate was analyzed using a spectrophotometer. The results, shown in Table 5, indicate that the removal percentages of the dye after regeneration with ZnO-RGO composite were 90.0%, 87.5%, and 80.0% for absolute ethanol, 1:1 aqueous ethanol, and 1:2 aqueous ethanol, respectively, with absolute ethanol achieving the highest removal percentage of 90.0%. This efficiency may be attributed to the hydrogen bonding strength of MB with alcohol molecules, which enhances MB solubility [140]. Overall, the results demonstrated that the removal percentage remained high after desorption, confirming the composite’s effectiveness in MB removal."

**Table 5:** Desorption of ZnO-RGO Composite

Desorption agent	Absolute ethanol	Aqueous ethanol (1:1)	Aqueous ethanol (1:2)
Removal, %	90.0	87.5	80.0

### Application

The three composites are used for adsorption of MB from a real sample (industrial drainage) under the optimum conditions (pH8, dose 0.15 g/100ml and 30 min for A1 and A2), while for A3 pH2, dose 0.1g/100 and 45 min. A standard addition method was used because the concentrations of all the assayed species were lower than the detection limit. 10 ppm MB was added to the solution, and then the residual concentrations of MB were calculated by the difference. As illustrated in Table 6, A3 nanocomposite achieves the highest removal percentage (97%) compared with A1 and A2 nanocomposites.

**Table 6:** Removal Percentage of MB Adsorption Using A1, A2 or A3

Removal, %			
Real sample	A1	A2	A3
Industrial drainage (1.3 mg /L)	88.5	89.3	97.0

## Conclusion

The three nanocomposites were successfully synthesized using three distinct methods. The first, a novel Leidenfrost green approach, was used to prepare the ZnO-RGO nanocomposite (A1), followed by the precipitation chemical method for the second composite (A2) and the physical mixing sonication method for the third (A3). Composites A1 and A2 underwent a reduction process due to the growth of ZnO nanoparticles on the RGO surface, while the A3 composite exhibited surface oxidation due to the mixing process between ZnO nanoparticles and RGO. A comparison of XRD results revealed that composite A3 showed the highest intensity peaks, indicating a higher degree of crystallinity compared to A1 and A2. The crystallite sizes were determined to be 16.7, 33.8, and 65.2 nm for A1, A2, and A3, respectively. TEM analysis showed ZnO nanoparticles distributed randomly on the RGO surface sheets, with ZnO in close contact with RGO. The surface areas for A1, A2, and A3 were 2.91, 7.29, and 1.90 m^2^/g, respectively.

For MB dye adsorption, A1 and A2 showed 85.5% and 87.5% effectiveness at pH 8, a dose of 0.15 g/100 mL, and a contact time of 30 min, while A3 showed 95% effectiveness at pH 2, a dose of 0.1 g/100 mL, and a contact time of 45 min. The isotherm study indicated that all three composites followed the Langmuir isotherm, with A1 showing the highest adsorption capacity of 104.5 mg/g, compared to A2 and A3, with 87.7 mg/g and 97.5 mg/g, respectively. The kinetic study showed that A3 had an R^2^ value greater than 0.93 across four study models, with the best fit to the pseudo-second-order model (R^2^ nearly 1). In contrast, A1 and A2 had R^2^ values less than 0.9 for all models except for pseudo-second-order, with values of 0.9998 for A1 and 0.9988 for A2."

## Data Availability

The authors declare that the data supporting the findings of this study are available within the paper.

## Abbreviations

SEM: Scanning electron microscopy
TEM: Transmission electron microscopy
XRD: X-ray diffraction analysis
FTIR: Fourier transform infrared spectroscopy
BET: Brunauer–Emmett–Teller
MB: Methylene blue
GO: Graphene oxide
RGO: Reduced graphene oxide
ZnO: Zinc oxide
A1: Composite 1
A2: Composite 2
A3: Composite 3
PZC: Point of zero charge
